# Titanium Allergy Caused by Dental Implants: A Systematic Literature Review and Case Report

**DOI:** 10.3390/ma14185239

**Published:** 2021-09-12

**Authors:** Pier Paolo Poli, Fábio Vieira de Miranda, Tárik Ocon Braga Polo, Joel Ferreira Santiago Júnior, Tiburtino José Lima Neto, Bárbara Ribeiro Rios, Wirley Gonçalves Assunção, Edilson Ervolino, Carlo Maiorana, Leonardo Perez Faverani

**Affiliations:** 1 Implant Center for Edentulism and Jawbone Atrophies, Maxillofacial Surgery and Odontostomatology Unit, Fondazione IRCCS Cà Granda Ospedale Maggiore Policlinico, University of Milan, 20122 Milan, Italy; carlo.maiorana@unimi.it; 2 Department of Diagnosis and Surgery, Division of Oral and Maxillofacial Surgery and Implantology, School of Dentistry, São Paulo State University—UNESP, Araçatuba 16015-050, SP, Brazil; fvmpatologia@yahoo.com.br (F.V.d.M.); tarikpolo@gmail.com (T.O.B.P.); tiburtinoneto@hotmail.com (T.J.L.N.); barbara.rios@unesp.br (B.R.R.); leonardo.faverani@unesp.br (L.P.F.); 3 Department of Health Sciences, Centro Universitário Sagrado Coração-UNISAGRADO, Bauru 17011-160, SP, Brazil; jf.santiagojunior@gmail.com; 4 Department of Dental Materials and Prosthodontics, School of Dentistry, São Paulo State University—UNESP, Araçatuba 16015-050, SP, Brazil; wirley.assuncao@unesp.br; 5 Department of Basic Sciences, School of Dentistry, São Paulo State University—UNESP, Araçatuba 16015-050, SP, Brazil; edilson.ervolino@unesp.br

**Keywords:** allergy, dental implants, hypersensitivity, peri-implant disease, titanium alloy

## Abstract

(1) Background: Hypersensitivity reactions to metals may arise in predisposed patients chronically exposed to metallic materials, including dental implants made of titanium alloys. The purpose of this article was to systematically review titanium allergy manifestations in patients treated with dental implants and report a clinical case; (2) Methods: A systematic electronic search was performed for articles published in the English language until July 2021. The following eligibility criteria were adopted: (1) Population: individuals undergoing titanium and/or titanium alloy implant-supported rehabilitations; (2) Exposure: peri-implant soft tissue reactions attributable to implant insertion; (3) Outcome: evidence of titanium allergy, diagnostic methods, and forms of resolution; (3) Results: The included studies, in summary, presented evidence that titanium should not be considered an inert material, being able to trigger allergic reactions, and may be responsible for implant failure. A 55-year-old male patient received 3 implants in the posterior region of the left mandible and presented an epulis-like lesion developed from the peri-implant mucosa. The immunohistochemical analysis of the biopsy specimen confirmed the initial diagnosis of allergic reaction to titanium; (4) Conclusions: Although the evidence is weak, and titanium allergy has a low incidence, hypersensitivity reactions should not be underestimated. A rapid and conclusive diagnosis is mandatory to prevent further complications.

## 1. Introduction

Titanium (Ti) is a widely used material in the manufacturing of dental implants. Ti plays a pivotal role in the osseointegration process by supporting the physicochemical attraction of osteoblast lineage cells. This leads to bone deposition that fills the space between the recipient bone and the implant surface [1]. The use of implants made of Ti in oral rehabilitations has been increasingly consolidated during the last decades also due to its biocompatibility [2]. This peculiar attribute of Ti allows its use in different clinical situations. In the medical field, metal alloys are widely used for the production of pacemakers, endoprostheses, and stents, and in dentistry they have shown excellent results due to excellent biocompatibility, strength, and capacity for osseointegration, and have been used for implants, crowns, bridges, and orthodontic appliances [3,4]. Furthermore, Ti implants have shown encouraging results in terms of survival rates in the long term [5,6,7].

In regards to Ti implant failures, they are generally linked to biologically related complications, more specifically recognized as peri-implant mucositis and peri-implantitis [8,9,10,11,12,13]. Besides disease entities induced by dental biofilms, another biological issue is related to allergic reactions to Ti. These are typically localized at the peri-implant soft tissues, either keratinized or not keratinized. Such hypersensitivity reactions to Ti are scarcely reported so far. Only a few cases have been reported in the literature concerning implant loss as a consequence of Ti allergy. Besides the dental field, primary sensitization to metal alloys has also been observed in the case of prostheses, endoprostheses, and stents, in addition to hypersensitivity reactions following pacemaker installations [3,14,15]. Given what has been analysed in the literature so far, there is no conclusive evidence that directly relates Ti allergy to dental implant failure. A literature review has shown the development of pyogenic granulomas and haemangiomas after dental implant installation [12,13,16]. Some of these cases exhibited metal particles in the histopathological analysis as a possible consequence of tribocorrosion. In other circumstances, hypersensitivity reactions resulted in peri-implant lesions accompanied in the vast majority by granulomas. Regrettably, specific tests or focused histopathological analyses are often not available, or are not carried out in the daily practice. Even when these diagnostic tools are available, due to clinical similarities with other postoperative complications including peri-implant mucositis and peri-implantitis, it is common that the physician or the dentist only rely on clinical and radiological evaluations without performing laboratory testing. Thus, in the diagnostic algorithm, hypersensitivity is not included in the differential diagnosis as a diagnostic hypothesis. This inevitably leads to a misdiagnosis that ultimately results in the removal of the affected implant [17,18].

In view of the above, the present study aimed to perform a comprehensive systematic review of the literature focused on Ti allergy related to dental implants. Moreover, a case of allergic reaction to Ti was also presented to better understand the clinical and histological manifestations of this adverse event.

## 2. Materials and Methods

The present study has been designed as a comprehensive systematic review performed according to the guidelines listed in The Preferred Reporting Items for Systematic Reviews and Meta-Analyses (PRISMA) statement [19]. A modified PICO format, namely the PEO (population, exposure, outcome) framework was applied [20]. The population consisted of individuals who were treated with Ti and/or Ti alloy implant-supported rehabilitations. The exposure was any peri-implant soft tissue reaction attributable to the insertion of dental implants. The assessed outcomes were: (1) the evidence of Ti allergy, (2) the methods used to reach a definitive diagnosis, and (3) the forms of resolution of the disease.

### 2.1. Search Strategy

The selection of all relevant studies was performed independently by three reviewers (F.V.M., T.P., and P.P.P.) and in three different databases: MEDLINE (PubMed), EMBASE, and The Cochrane Library. All human studies published in the English language from 1990 reporting on dental implant allergy were considered eligible for inclusion. The latest electronic search was undertaken in July 2021. The following combinations of Medical Subject Headings (MeSH) and entry terms were used in the electronic search: “(dental implants [MeSH] AND (allergy OR allergic reaction OR hypersensitivity [MeSH]))”. A fourth author (L.P.F.) reviewed the workflow of the search strategy. The articles were selected by title and abstract and in accordance with the inclusion and exclusion criteria.

### 2.2. Selection Criteria

In the study protocol, inclusion and exclusion criteria for study eligibility were defined. Due to a reduced number of randomized controlled clinical trials (RCTs), all clinical study types on human subjects were included in the present review, including RCTs, controlled clinical trials, prospective and retrospective studies, case series, and case reports. Conversely, animal or in vitro studies, as well as review articles, were excluded. During the collection of data, any disagreement was discussed and resolved. At the end of the screening process, no disagreement among the authors regarding the results was found. The interexaminer Cohen’s kappa coefficient (κ) was used to evaluate the inter-rater agreement between the authors in the selection of the articles for each database. After study selection, a high level of agreement among independent examiners was observed in all databases: MEDLINE (PubMed): (κ = 0.94); EMBASE: (κ = 0.9); The Cochrane Library: (κ = 1).

## 3. Results

**Figure 1:**
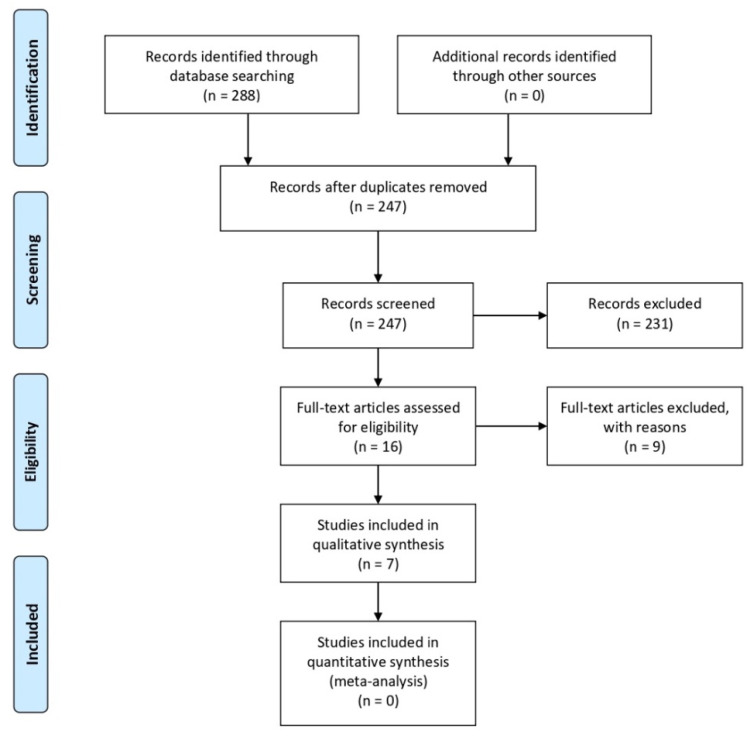
Flow diagram according to the PRISMA guidelines summarizing the systematic screening process.

The PRISMA flow diagram is illustrated in Figure 1.

The search strategy in the selected databases yielded a total of 288 articles. Overall, 223 articles were excluded after the evaluation of the title and abstract according to the inclusion and exclusion criteria. There were 41 duplicate references, and 8 articles were neither accessible from MEDLINE (PubMed) nor retrievable from the corresponding authors. Thus, 16 articles were finally available for the full-text examination [2,18,21,22,23,24,25,26,27,28,29,30,31,32,33,34]. The evaluation of these articles in full resulted in the selection of seven studies [18,23,25,29,32,33,34] for the qualitative analysis and none for the quantitative analysis (Figure 1).

Of the seven studies selected for the qualitative analysis [18,23,25,29,32,33,34], three studies were prospective studies or case series [23,25,32], while the remaining four were case reports [18,29,33,34] (Table 1 and Table 2). A total of 401 patients were evaluated, 105 males and 296 females. Three of the selected studies did not specify the implant system used and the number of implants, nor did they specify the location of the implants in relation to the surgical site, the radiological aspects, the use of medications, and the histopathological analysis [23,25,32]. Differently from the other two studies [23,25], the study by Hosoki et al. [32], illustrated the treatment that had been performed. The study by Muller et al. [25], did not present data on the clinical manifestations of the allergic reaction. The total number of patients who presented clinical manifestations of allergic reactions following implant therapy were 25 subjects divided into six studies [18,23,29,32,33,34].

### 3.1. Implant Systems

Three of the included studies [18,29,33] presented details concerning the implant system that has been used. In the study by Du Preez et al. [18], two implants were cylindrical units (GMI, Southern Implants (Pty) Ltd., Centurion, South Africa), two were single-stage compression implants (LIBB, Southern Implants (Pty) Ltd., South Africa), and two were Branemark-like designed implants (IBS, Southern Implants (Pty) Ltd., Centurion, South Africa). In the study by Olmedo et al. [29], two different implant systems were used. In the first case, a single Ti grade 4 acid-etched surface (Titantec, Proaltec S.A., Buenos Aires, Argentina) was placed; in the second case, three Branemark-like designed implant were used, but the manufacturer of the implants was unknown. In the study by Hosoki et al. [33], two implants (Fixture MicroThread system, Astra Tech Implant System, Mölndal, Sweden) were used, characterized by a rough surface (TiOblast) produced by blasting with titanium dioxide particles.

### 3.2. Clinical Manifestations

Clinical manifestations have been reported in six studies [18,23,29,32,33,34]. In the case reported by du Preez et al. [18], the patient presented swelling in the peri-implant tissues, in the submental region, and at the labial sulcus, associated with pain and hyperaemia in the peri-implant tissues, without pus discharge and necrosis. Olmedo et al. [29] reported specific characteristics for each treated case. In the first report, following two postoperative months, the patient presented with a lesion measuring 1 cm × 1 cm × 0.6 cm with a smooth and bright surface, red and bleeding on palpation; in the second case, the patient showed a vestibular sessile lesion distally to the maxillary left lateral incisor, measuring 0.6 cm × 0.5 cm × 0.4 cm, with a reddish and irregular aspect. In the work by Sicilia et al. [23], considering the test group composed of 35 individuals, a total of 16 subjects had clinical symptoms and/or implant loss. The remaining 19 patients had a history of other allergies or predisposing factors for implant failure. In the study by Hosoki et al. [33], the authors observed eczema two years after implant installation, triggered by orthopaedic surgery. The previous authors published an additional report in 2018 [32], where four more patients presented with allergic manifestations of eczema and local reactions. Borgonovo et al. noticed swelling and redness in peri-implant tissues and bleeding, a probe depth of 6 mm in the buccal side and 5 mm in the lingual side, with high mucosa sensitivity and implant exposure [34].

### 3.3. Imaging Tests

Only 3 studies reported radiographic manifestations [18,29,34]. In the case reported by du Preez et al. [18], the radiographic evaluation showed ill-defined radiolucent areas with ragged margins at the apices and at the lateral aspects of the implants. Olmedo et al. [29] observed no bone loss in the first case, whereas in the second case, a cup-shaped bone resorption was found. Borgonovo et al. showed a bony defect with a crater-like shape around the first molar implant, and cervical decay on teeth and vertical bone loss involved the new implants; the process of external resorption affected the teeth up to the canine [34].

### 3.4. Medications

The only study that reported the use of antibiotics following detection of clinical manifestations was the case reported by du Preez et al. [18], who prescribed amoxicillin 500 mg every 8 h for five days, and additional 400 mg metronidazole every 8 h, however the exact duration of the therapy was not specified.

### 3.5. Treatment

Overall, five studies reported details on the treatment that had been performed [18,29,32,33,34]. In the study by du Preez et al. [18], affected implants were removed, followed by debridement and peri-implant biopsy. Similarly, Olmedo et al. [29] described the surgical removal of the peri-implant mucosa lesion (biopsy) in both cases. The removal of implants was also the treatment of choice in both studies published by Hosoki et al. [32,33]. In the study by Hosoki et al. [33] in 2016, the orthopaedic screws were also removed in addition to the implants. Borgonovo et al. described the titanium implant removal after nine months when allergic symptoms disappeared; five one-piece zirconia implants were inserted, four in the anterior jaw and one in the right molar region [34].

### 3.6. Histopathological Analysis

Histopathological analyses were described in three studies [18,29,34]. In the study by du Preez et al. [18], 8 samples revealed foci of subacute inflammation, moderate chronic inflammation, lymphocytes, plasma cells, concomitant fibrosis histiocytes, while 7 samples showed granulation tissue and giant cells. In the cases reported by Olmedo et al. [29], the first case showed intense mixed inflammatory infiltrate, vascular proliferation, and abundant macrophages. Numerous metal-like particles have been identified, included within macrophages and perivascular regions. In the second case, proliferation of fusiform and round mesenchymal cells, intense vascularization, and numerous multinucleated giant cells with isolated metal particles were observed. Borgonovo et al. mentioned that the biopsy was performed by taking a sample of cortical and medullary tissue. The results did not show any kind of bone lesion or disease [34].

### 3.7. Additional Tests

In the study by Olmedo et al. [29], the identification of metals was performed by emission spectroscopy; however, the authors did not identify the type of metal, such as titanium, due to the small mass of the sample. In the work by Sicilia et al. [23], skin tests (type I hypersensitivity), and epicutaneous tests (type IV hypersensitivity) were carried out. Muller et al. [25] used the MELISA^®^ test and patch test. The results reported by du Preez et al. [18], showed type IV late hypersensitivity as evidence of true allergic reaction or hypersensitivity to dental implants. In the cases reported by Olmedo et al. [29], the first patient was diagnosed with pyogenic granuloma, and after surgical excision, no recurrences were observed. In the second case, a diagnosis of peripheral giant cell granuloma was made. In the work by Sicilia et al. [23], 25.7% of the subjects in the test group had positive results for titanium allergy following a skin or epicutaneous test. In the control group, all the patients were negative for titanium allergy. In the study by Muller et al. [25], of the 56 patients tested in MELISA^®^, 21 (37.5%) were positive, 16 (28.6%) ambiguous, and 19 (33.9%) negative to Ti. The authors concluded that Ti can induce hypersensitivity and should not be considered an inert material. In both studies published by Hosoki et al. [32,33], patch tests were performed to analyse 28 different types of metals. The first study suggested that the patient was sensitized by the orthopaedic procedure and developed Ti allergy thereafter, while in the second study, 80.4% of the patients were positive for at least one metal, and 4 patients were positive for Ti. Borgonovo et al. mentioned that the MELISA test (Memory Lymphocyte Immunostimulation Assay) was performed and confirmed titanium hypersensitivity, and the standard blood tests revealed an increased number of eosinophils. In addition, the bacterial culture was negative [34].

### 3.8. Case Report

**Figure 2:**
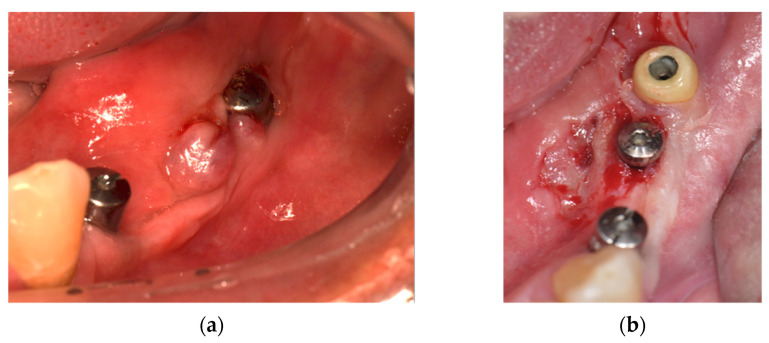
Clinical situation at baseline. (**a**) Intraoral view of the peri-implant lesion after 7 days of healing; (**b**) Intraoral view of the peri-implant soft tissues after biopsy of the lesion.

The treatments performed in the present case followed the Declaration of Helsinki on medical protocol and ethics. A 55-year-old male presented with no underlying diseases such as diabetes or hypertension, not in therapy with any type of medication that would interfere with tissue or bone metabolism, and with no history of allergy to metals or precious jewels. Patient was referred to the dental clinic seeking a fixed implant-supported rehabilitation in the lower left posterior mandible. Overall, three Ti dental implants (commercially pure titanium-Conexão implants^®^, Arujá, SP, Brazil: 3.75 × 11 mm (region of the first premolar); 4 × 7 mm; 4 × 7 mm (region of the second premolar and first molar respectively)) were placed with a submerged approach. After 5 months from implants placement, the re-entry procedure was performed to uncover the implants and connect the Ti healing abutments. After a healing period of seven days, an epulis-like, reddish lesion was observed, with oedematous soft tissues covering the healing abutments connected to the most distal implants (Figure 2a). The first approach was a biopsy of the lesion (Figure 2b).

**Figure 3:**
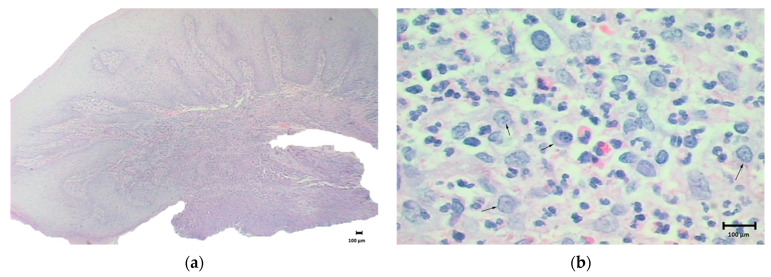
Histological analysis of the lesion. (**a**) Histological image showing inflammatory process, magnification: 10×, staining: hematoxylin and eosin; (**b**) Histological image showing polymorphonuclear cells (black arrows), magnification 100×, staining: hematoxylin and eosin.

The histological analysis revealed an inflammatory infiltrate populated by eosinophils and polymorphonuclear neutrophils (Figure 3a,b) suggesting an allergic reaction.

**Figure 4:**
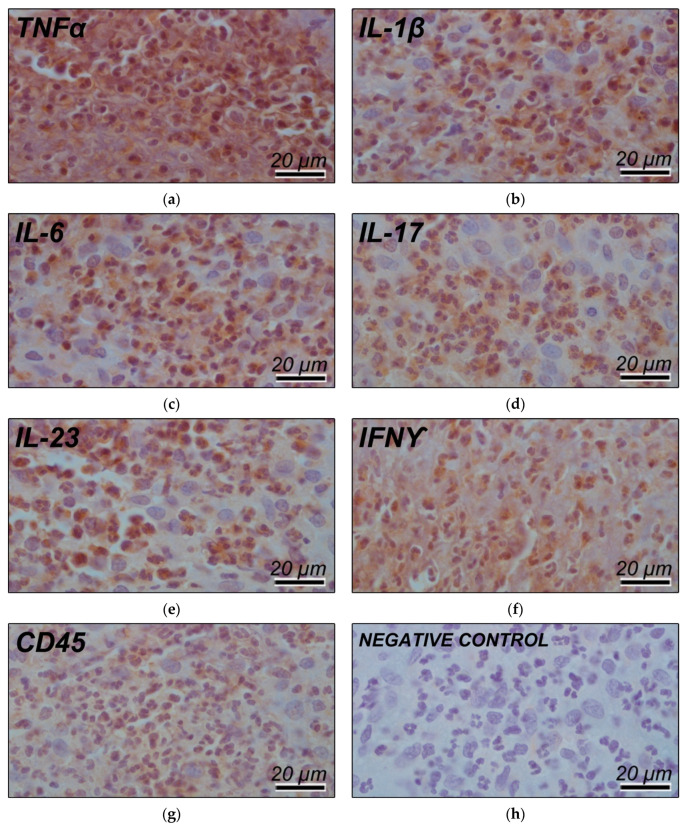
Immunohistochemical panel of (**a**) TNFα, (**b**) IL1, (**c**) IL6, (**d**) IL17, (**e**) IL23, (**f**) IFNγ, (**g**) CD45 proteins, and (**h**) negative control, showing intense areas of allergic response labelling. Magnification 100×, staining: Harris’s hematoxylin.

Given this histopathological feature, panels of immunohistochemical markers for TNF-alpha, IL-1, IL-6, IL-17, IL-23, IFN-gamma, and CD-45 were performed, confirming intense areas of inflammatory marker response typical of allergic reaction (Figure 4a,h).

**Figure 5:**
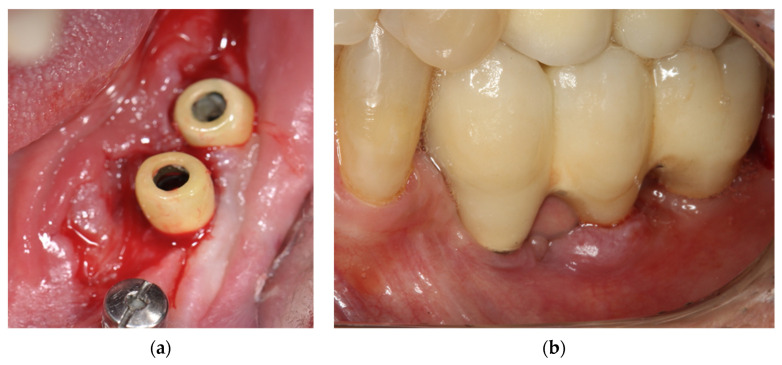
Clinical situation during the healing phase. (**a**) Intraoral view of the peri-implant soft tissues and the plastic healing caps connected to the distal implants after 10 days; (**b**) Intraoral view at the 5-year follow-up recall.

Interestingly, in correspondence to the mesial implant originally surrounded by a thick layer of keratinized mucosa, no lesions were detected. It may be speculated that the presence of a keratinized tissue layer able to protect the underlying tissues from a potential metal allergy prevented the onset of hypersensitivity reactions. After 7 days from the biopsy, a recurrence of the epulis-like lesion was observed in the same anatomical site. Therefore, conventional healing abutments were replaced with plastic implant abutments covered with ceramic. After 10 days, regression of the lesion was noted (Figure 5a). The definitive implant-supported screw-retained fixed dental prosthesis was finally screwed to the implants. Also, the patient was subjected to an epicutaneous allergenic test, the Patch test. Ti and some metallic alloys were investigated, resulting in a cutaneous allergy to Ti compounds, which confirmed the diagnostic hypothesis.

Regular clinical and radiological follow-up recalls were scheduled thereafter. At the 5-year clinical and radiographic examination, no recurrence of the lesion was observed (Figure 5b).

## 4. Discussion

The rationale of the present systematic review was to summarize the current evidence related to Ti allergy and consequent failure of dental implants. The aim was also to bring light the possible failures in implant dentistry that are often not investigated and not diagnosed correctly in the daily practice. The results that emerged from the search strategy indicated that only a few studies are currently available in the literature, and thus the evidence on this topic is weak. The lack of clinical investigations using accurate diagnostic methods to evaluate biological complications caused by allergic reactions to metals may underestimate the actual failure rate found in the literature. The work by Sicilia et al. [23] strengthened the possible relation between metal allergy observed after installation of Ti fixtures and implant failures. This relation has been supported by allergy tests to several metals, including Ti. In 5 cases, patients presented postoperative symptoms attributable to metal allergy, such as oedema of the face and of the glottis, mucositis, and hyperplasia. In 3 reports, the tests were positive for Ti allergy, and the treatment was the delivery of the prosthesis, thereby reducing the contact of the mucosa with the Ti components. Another option was to replace Ti implants with zirconia implants. No further complications were observed at the 5-year follow-up. The aforementioned clinical manifestations were similar to those reported in the case described herein. Indeed, direct contact with the metal components caused biological complications in the peri-implant mucosa during the preprosthetic phases. A significant clinical improvement was observed when plastic prosthetic components covered by porcelain were installed in the affected site. This stratagem avoided the direct contact of the peri-implant mucosa with the metal. In the study by Sicilia et al. [23], additional 5 cases had biological complications of unexplained origin, leading to implant loss. These failures are often not correctly diagnosed in the literature due to a lack of diagnostic tests able to confirm the relation between peri-implant lesions and Ti allergy. Some of these failed implants were replaced by zirconia implants if patients were still willing to receive implant-supported restorations. In all such cases, the tests were positive for Ti allergy. This confirmed the relationship between peri-implant mucosal reactions and allergy to metals such as Ti.

The case reported by du Preez et al. [18] deserves additional attention. In the postoperative period following implant placement, the patient started feeling discomfort. The medication was changed, and antibiotic therapy with amoxicillin was complemented with metronidazole. Nevertheless, the symptoms worsened, including oedema in the submental region and in the labial sulcus, localized pain, and surrounding hyperaemia of the soft tissues. In the panoramic radiograph, evidence of pathological peri-implant bone loss was observed. Implant removal was performed, and the biopsy of peri-implant material suggested a foreign body reaction, with a typical inflammatory infiltrate and multinucleated giant cells. In both studies published by Hosoki et al. [32,33], removal of the implants was recommended; however, no biopsy of the peri-implant tissues was performed for histological evaluation. The study by Muller et al. [25] was not directly related to clinical complications; however, it may be considered useful to reinforce and suggest, through the MELISA^®^ test, the possible hypersensitivity reaction that Ti may cause.

In summary, reports on peri-implant mucosal reactions generally advocated the removal of the implants involved as a resolutive therapy. When available, the histopathological analysis commonly revealed the presence of macrophages and cells responsible for allergic reactions. These features, together with the presence of eosinophils, have also been observed in the present case. It is worth mentioning that, sometimes, it is possible to notice “metal-like” unstained particles located in the peri-implant tissues. Spectroscopic techniques identified this foreign material as Ti particles or particles originated from other metals belonging to dental implant components [7,12,13,29].

The question of whether the indication of implant removal might be the first therapeutic option in the presence of peri-implant mucosal reaction has still yet to be confirmed. Considering what has been found in the articles included in the present review, and according to the treatment approach performed in the case reported herein, a therapeutic algorithm may be suggested. A possible treatment strategy may include: (1) biopsy of the peri-implant soft tissues including the lesion; (2) avoid contacts between the peri-implant soft tissues and Ti by means of metal-free prosthetic components; (3) perform skin and enzyme immunoassays to verify allergies to Ti and other metals; (4) consider peri-implant plastic surgery techniques including subepithelial connective tissue grafts or free-gingival grafts to improve the peri-implant soft tissues, particularly in those situations presenting with a lack of keratinized mucosa.

## 5. Conclusions

Despite the low level of evidence, hypersensitivity to Ti and its alloys can occur and should not be underestimated. Clinical manifestations and histological features are typical of allergic reaction and may help the clinician in the differential diagnosis. Lesions attributable to Ti allergy should therefore be recognized and diagnosed early in order to enhance both treatment and prognosis.

## Figures and Tables

**Table 1:** Characteristics of the included studies and demographic data.

Author	Study Type	Sample Size	Mean Age (Years)	Sex	Total Implants	Position	Implant Data
Borgonovo et al. [34]	Case report	1	56	Female	4	Mandible	Implants in the region of mandibular teeth (right canine up to first molar)
du Preez et al. [18]	Case report	1	49	Female	6	Mandible	GMI, LIBB, IBS, (Southern Implants)
Olmedo et al. [29]	Case report	2	69.5	Females	3	Case 1: mandible	Case 1: Titantec 4.1 mm × 10 mm
						Case 2: maxilla	Case 2: two implants in the region of elements 22 and 23; 4.1 mm × 11.5 mm (unknown brand)
Sicilia et al. [23]	Case series	Test: 35	Test: 50.2	Test: 10 males; 25 females			
		Control: 35	Control: 47.69	Control: 16 males; 18 females			
Muller et al. [25]	Clinical and laboratory study	56	53.8	17 males; 39 females			
Hosoki et al. [33]	Case report	1	69	Male	2	Right lower molars	Fixture MicroThread system, Astra Tech
Hosoki et al. [32]	Clinical study	270	53.9	61 males; 209 females			

**Table 2:** Overview of the characteristics of the lesions, the type of medications and treatments, additional tests and diagnosis.

Author	Clinical Features	Imaging Features	Medications	Treatment	Histopathology	Other Tests	Results
Borgonovo et al. [34]	Swelling and redness in peri-implant tissues and bleeding and a probing depth of 6 mm, bucally and 5 mm lingually, high mucosa sensitivity and implant exposure.	a bony defect with a crater-like shape around first molar implant and cervical decay on teeth and vertical bone loss involved the new implants and the process of external resorption affected the teeth up to the canine		Removal of the titanium implant, after nine months, when allergic symptoms disappeared, five one-piece zirconia implants were inserted, four in the anterior jaw and 1 in the right molar region	Biopsy was performed by taking a sample of cortical and medullary bone to check for bone disorder. the result did not show any kind of bone lesion or disease.	A standard blood tests revealed an increased number of eosinophils. MELISA (Memory Lymphocyte Immunostimulation Assay) test was performed and confirmed titanium hypersensitivity. The bacterial culture was negative.	During the follow-up period, the patient did not refer to any symptoms of peri-implantitis or other problems, and after 18 months from surgery, the clinical-radiographic exams showed the success of the metal-free implant prosthetic rehabilitation.
du Preez et al. [18]	Swelling in peri-implant tissues, swelling in the submental region and lip crease, pain, hyperaemia in peri-implant tissues (no pus and no necrosis)	irregular radiolucent areas at the apex and sides of the implants	Postoperative: Amoxicillin 500 mg 8/8 h and ibuprofen 400 mg 8/8 h; After clinical manifestations: Metronidazole 400 mg	Implant removal, debridement and peri-implant biopsy	8 samples revealed foci of subacute inflammation, moderate chronic inflammation, lymphocytes, plasma cells, concomitant fibrosis histiocytes; 7 samples revealed granulation tissue and giant cells.	None	Type IV (late) hypersensitivity diagnosis, evidence of a true allergy or hypersensitivity to dental implants
Olmedo et al. [29]	Case 1: two postoperative months, lesion measuring 1 × 1 × 0.6 cm, smooth and bright red surface and bleeding on palpation	Case 1: no bone loss	chlorhexidine 2% postoperatively	Case 1: surgical removal of the lesion and curettage, implant was maintained (biopsy)	Case 1: intense vascular proliferation, mixed inflammatory infiltrate and abundant macrophages. Numerous metal-like particles have been identified, including within macrophages, perivascular region	Identification of metals by emission spectroscopy: did not identify titanium due to small sample	Case 1: pyogenic granuloma diagnosis and 4-year-old patient with no recurrence
	Case 2: distal vestibular sessile lesion around implant 22 measuring 0.6 × 0.5 × 0.4 cm, red and irregular	Case 2: cup-shaded bone loss		Case 2: surgical removal and buccal bone curettage	Case 2: proliferation of fusiform and round mesenchymal cells, intense vascularization, and numerous multinucleated giant cells. Isolated metal particles		Case 2: Diagnosis of peripheral giant cell granuloma, 2-year-old control patient without injury
Sicilia et al. [23]	16 individuals had clinical symptoms and/or implant loss. 19 individuals had a history of other allergies or predisposing factors for implant failure					Skin tests (type I hypersensitivity); Epicutaneous tests (type IV hypersensitivity)	25.7% tested positive for titanium allergy in the test group. (positive for skin test or epicutaneous test). In the control group, 100% tested negative.
Muller et al. [25]						Test MELISA^®^ and patch test	It was not specific to the dental implant group. Test patch was negative for Ti in all cases; MELISA^®^ test was positive for Ti in 37.5% cases, and 21.4% cases showed reaction to Ni. Morphologically, the analyses confirmed the presence of lymphoblasts and in the positive results Ti was observed in the macrophages. Ti may induce hypersensitivity and should not be considered an inert material
Hosoki et al. [33]	Eczema 2 years after implant placement, triggered by orthopaedic surgery	No changes		Removal of orthopaedic screws (50% improvement), removal of metal restorations and oral prostheses (30% improvement), removal of implants		Patch Test for 28 metal types	Study concluded that the patient was possibly sensitized by orthopaedic surgery and developed implant allergy to Ti
Hosoki et al. [32]	4 patients had allergic manifestations of eczema and local reactions			3 of the patients who presented reactions underwent implant removal		Patch Test for 28 metal types	217 patients (80.4%) positive for at least one metal. Of the 16 patients with signs of allergy after implant placement, 11 of these were positive for other metals and 4 were positive for Ti

## Data Availability

No new data were created or analyzed in this study. Data sharing is not applicable to this article. The corresponding author remains available for any further clarification.
